# Improving the Linkages between Air Pollution Epidemiology and Quantitative Risk Assessment

**DOI:** 10.1289/ehp.1103780

**Published:** 2011-08-04

**Authors:** Neal Fann, Michelle L. Bell, Katy Walker, Bryan Hubbell

**Affiliations:** 1 Office of Air Quality Planning and Standards, U.S. Environmental Protection Agency, Research Triangle Park, North Carolina, USA; 2 School of Forestry and Environmental Studies, Yale University, New Haven, Connecticut, USA; 3 Health Effects Institute, Boston, Massachusetts, USA

**Keywords:** epidemiology, health impact assessment, risk assessment

## Abstract

Background: Air pollution epidemiology plays an integral role in both identifying the hazards of air pollution as well as supplying the risk coefficients that are used in quantitative risk assessments. Evidence from both epidemiology and risk assessments has historically supported critical environmental policy decisions. The extent to which risk assessors can properly specify a quantitative risk assessment and characterize key sources of uncertainty depends in part on the availability, and clarity, of data and assumptions in the epidemiological studies.

Objectives: We discuss the interests shared by air pollution epidemiology and risk assessment communities in ensuring that the findings of epidemiological studies are appropriately characterized and applied correctly in risk assessments. We highlight the key input parameters for risk assessments and consider how modest changes in the characterization of these data might enable more accurate risk assessments that better represent the findings of epidemiological studies.

Discussion: We argue that more complete information regarding the methodological choices and input data used in epidemiological studies would support more accurate risk assessments—to the benefit of both disciplines. In particular, we suggest including additional details regarding air quality, demographic, and health data, as well as certain types of data-rich graphics.

Conclusions: Relatively modest changes to the data reported in epidemiological studies will improve the quality of risk assessments and help prevent the misinterpretation and mischaracterization of the results of epidemiological studies. Such changes may also benefit epidemiologists undertaking meta-analyses. We suggest workshops as a way to improve the dialogue between the two communities.

Air pollution epidemiology plays an integral role in both identifying the hazards of air pollution to human health and informing the design and implementation of air quality policy (Greenbaum et al. 2001). A large and growing body of epidemiological studies has helped characterize for policy makers the link between ambient air pollution and the risk of an array of adverse health outcomes. In particular, those studies that have observed a relationship between both long-term exposure to particulate matter ≤ 2.5 μm in aerodynamic diameter (PM_2.5_) and premature death and between short-term ozone exposure and morbidity impacts have provided key empirical evidence in support of air quality standards (Ito et al. 2003; Pope et al. 2002). The findings of these and other epidemiological studies have informed critical policy decisions regarding the appropriate levels of the National Ambient Air Quality Standards for the six common “criteria pollutants” in the United States [U.S. Environmental Protection Agency (EPA) 2007b] and the levels of the World Health Organization (WHO) air quality guidelines (WHO 2005) in Europe.

No less important, but perhaps not as broadly recognized, is the role of air pollution epidemiology in supporting quantitative risk assessments—principally by providing the risk coefficients that relate air quality changes to the probability of a variety of adverse health outcomes, including premature death, hospital visits, and acute respiratory symptoms, among many others. In general, risk assessments aim to answer one of two types of policy questions. First, what is the total public health burden associated with exposure to air quality levels above some background level in terms of the number of excess cases of premature death or illness? Examples of this type of analysis include a report by Cohen et al. (2004) that quantified about 800,000 premature PM_2.5_-related deaths per year in urban areas globally and a global burden assessment by Anenberg et al. (2010) that estimated approximately 4.2 million premature deaths per year attributable to anthropogenic PM_2.5_ and ozone. A second and related question is, what would be the impact on human health of incremental changes in air quality due to a proposed policy? As an example, the U.S. EPA assessment of the benefits of the Clean Air Act Amendments of 1990 [Clean Air Act Amendments 1990; §109(b)(1)] estimated approximately 230,000 PM_2.5_ and ozone-related premature deaths were avoided beginning in 2020 due to the implementation of the Clean Air Act Amendments (U.S. EPA 2011). In Europe, a WHO analysis of transboundary air pollution found that a climate policy incorporating “maximally technically feasible reductions” could reduce the number of ozone-related deaths by approximately 8,000 per year (WHO 2008).

These types of quantitative risk assessments are frequently performed in a cost–benefit framework where the health impact estimates expressed as an economic value can be substantial (U.S. EPA 2010). The risk assessment accompanying a recent rule affecting coal-burning electrical-generating units used risk coefficients drawn from two long-term mortality studies based on the American Cancer Society and Harvard Six Cities cohorts (Laden et al. 2006; Pope et al. 2002) to estimate a change in PM_2.5_-related premature mortality of 14,000 and 36,000; the analysis estimated the economic value of these and other health and welfare benefits to be between $120 to $270 billion U.S. dollars (3% discount rate, 2006 dollars) (U.S. EPA 2010). In many cases, the findings of these risk and benefits assessments are broadly cited by the media and used by policy makers to justify significant changes in air quality policy (New York Times 2010; U.S. Congress 2010).

Air pollution epidemiology and quantitative risk assessment are sometimes thought to be distinct disciplines with slightly different aims—epidemiology being concerned with hypothesis testing and hazard identification and risk assessment with adapting these findings to answer policy questions. This commentary contends that these two disciplines in fact share a number of interests and that much can be gained by their tighter integration. Perhaps the most obvious of these interests is a common desire to ensure that the results of epidemiological studies are used appropriately in risk assessments [Hubbell et al. 2009; NARSTO (formerly North American Research Strategy for Tropospheric Ozone) 2010]. Although risk assessors seek to use the best available data in their analyses, epidemiologists wish for their data not to be misused and their findings not to be misinterpreted. An extensive history of scientists and institutions offers guidance on how this relationship should work—how epidemiological data might be used responsibly and effectively in air pollution risk assessment (National Research Council 1985, 1990, 1997, 2002, 2008; Neutra et al. 2006; WHO 2000). We add to this literature by suggesting a series of modest changes to the presentation of the data and to the findings of epidemiological studies that will encourage the accurate use of this information in quantitative risk assessments and, at the same time, benefit those epidemiologists who undertake meta-analyses. We also suggest that an improved dialogue between these two communities might highlight areas for future research relevant to both communities.

## Using the Findings from Epidemiology Studies in Quantitative Risk Assessments

Risk assessments generally apply a health impact function combining *a*) risk estimates from the epidemiology literature that relate air quality changes and health outcomes, *b*) modeled or observed air quality changes to characterize exposure, *c*) the population at risk, and *d*) baseline health status (prevalence and incidence of disease) of that population. For the key data necessary to specify this health impact function, analysts look to epidemiological studies for a quantitative measure (often referred to as an “effect estimate”) relating changes in air quality to changes in health risk.

As noted above, there is an extensive body of literature that broadly describes the appropriate use of epidemiological evidence in health risk assessment. Among the most important considerations are *a*) accounting for differences in the demographic profile and air pollution exposure of the evidentiary and target populations, *b*) avoiding double counting of impacts, and *c*) characterizing the sensitivity of the results to key input parameters. Many (although by no means all) of the uncertainties inherent in risk assessments are influenced greatly by the methodological choices of the epidemiological study—which the risk assessment should account for, or at least acknowledge, to the extent possible with the reported data. However, obtaining the necessary information from published epidemiological studies to fulfill these guidelines is sometimes challenging. In many cases, study authors may not consider these data to be central to the hypothesis being tested and so will choose not to report this information—perhaps not realizing how these data might contribute to a more appropriate use of the study findings. Below we discuss the aspects of epidemiological studies that, although not always reported, are important to well-designed risk assessments. We also describe instances in which the data reported in epidemiological studies directly influenced the design of health impact analyses and subsequent policy decisions.

***Key attributes of epidemiological studies relevant to risk assessments.* Effect estimates.** Among the first steps in the risk assessment is properly accounting for the model specification of the epidemiological study within the health impact function. Although the use of Poisson or logistic regression in a study is generally very clear and easily accounted for in the health impact function, there are a variety of other data useful to the risk assessor that, although perhaps not readily accessible, are important to the quantification and characterization of impacts. For example, many epidemiological studies conduct analyses of the sensitivity of risk coefficients to alternate model specifications, which can also provide extremely valuable insights for risk assessments and meta-analyses, although researchers may not fully describe this information. Such sensitivity analyses might include adjustment for confounders (e.g., other pollutants) or effect modifiers (e.g., demographic variables), information that can be incorporated into risk assessments. More routine numerical presentation of uncertainty for risk coefficients and other variables (e.g., *t*-statistics, *p*-value, 95% confidence interval, standard error of central estimates) would also be useful to risk assessments. Where studies consider the combined (or synergistic and antagonistic) impacts of exposures to two or more pollutants, or temperature and one or more pollutants, a variance–covariance matrix can allow the risk assessor to quantify confidence intervals from a joint uncertainty distribution. Finally, null or not statistically significant results, although less likely to be published, may still prove useful to those performing risk assessments and meta-analyses and warrant closer attention than they currently receive.

**Figure 1:**
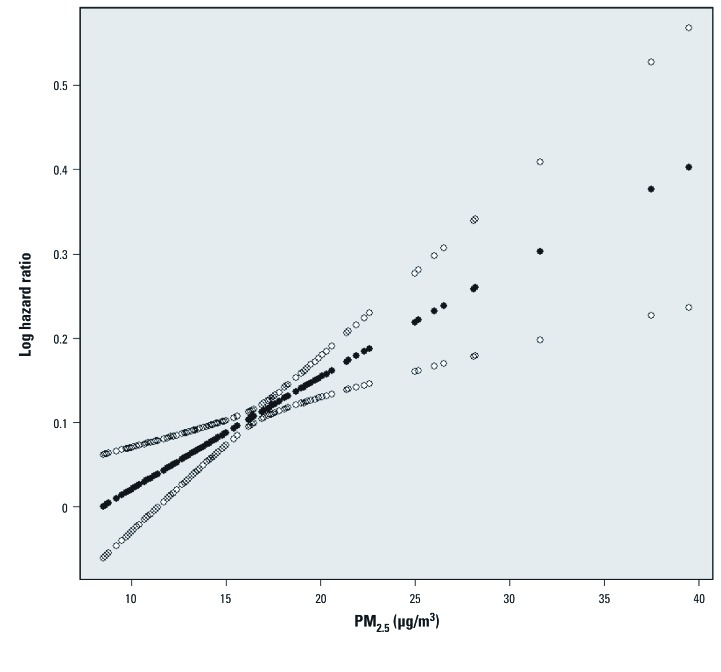
The estimated concentration–response relationship between PM_2.5_ and the risk of death in the Harvard Six Cities Study, using a penalized spline with 18 knots (solid circles) and pointwise 95% confidence intervals (open circles). Reproduced from Schwartz et al. (2008) with permission from *Environmental Health Perspectives*.

Although it is generally true that those performing either risk assessments or meta-analyses would prefer more detailed information regarding key data and assumptions, the study author’s choice of how to present this information can greatly influence its interpretability to different audiences. In many cases, the presentation of risk estimates in a comprehensive table (for example, see Bell and Dominici 2008, their Table 5) that account for alternate model specifications and effect modifiers will provide the details necessary to inform a risk assessment. However, data-rich graphics can complement the presentation of certain tabular data and yield unique insights. For example, the U.S. EPA recently evaluated the long-term PM–mortality literature to consider the empirical basis for a threshold in the concentration–response relationship. The U.S. EPA found useful the graphics depicting the concentration–response curve and 95th percentile confidence interval over the range of the observed data [for example, see Figure 1 (Schwartz et al. 2008)]. These illustrations demonstrated the relationship between the width of the confidence intervals around the mean estimate at various air quality levels and the density of the observed air quality data—helping the U.S. EPA to evaluate the plausibility of a concentration threshold in the PM–mortality relationship.

**Air quality.** A major analytical challenge facing risk assessments is ensuring that their treatment of modeled or observed air quality changes or exposure estimates is compatible with the treatment of air quality in the epidemiological study. This can relate to the composition and relative levels of pollution mixtures over space and time, methods used to estimate exposure, or the characterization of pollutant exposures. An example is the air quality metric used to assess short-term ozone-related premature mortality. In its 2008 assessment, the U.S. EPA relied on a national time-series study and three meta-analytic studies (Bell et al. 2004, 2005; Ito et al. 2005; Levy et al. 2005). Each study related mortality risk to a 10-ppb change in one or more ozone metrics, including the 24-hr mean, the 1-hr daily maximum, and the 8-hr daily maximum. Consequently, when performing its risk assessment, the U.S. EPA converted each risk coefficient based on a 24-hr mean or a 1-hr maximum into one based on an 8-hr maximum, assuming a constant ratio between each pair of metrics (U.S. EPA 2007a). This process introduces uncertainty to the analysis (Anderson and Bell 2010). Although the availability of air quality data affected the selection of metrics in each study, presentation of results from multiple metrics would have mitigated this uncertainty for both risk assessors as well as those performing meta-analyses.

Likewise, PM_2.5_ composition varies across locations and seasons and may affect its toxicity. Recent literature suggests that some chemical components and sources may have stronger effects than others and that differences in effect estimates across cities or seasons may relate to the chemical composition of particles (Bell et al. 2009; Franklin et al. 2007; Laden et al. 2000; Lippmann et al. 2006; Peng et al. 2009; Zanobetti et al. 2009). Presentation of information regarding the chemical speciation of PM_2.5_ mass, if available, is thereby valuable to risk assessments in the characterization, if not quantification, of results. This information could permit assessors to down- or up-weight results (qualitatively or quantitatively) based on differences (or similarities) in the particle composition between the target and evidentiary populations.

Risk assessments rarely have the opportunity to rely on risk coefficients from epidemiological studies in which the temporal and spatial variability in air quality is fully consistent with that of the policy scenarios being analyzed. For example, risk assessments frequently apply projected air quality values, sometimes resulting in a spatial distribution and temporal pattern of air pollution that diverges considerably from the epidemiological study; this is an especially important issue for risk assessments transferring risk coefficients from one region of the world to another, where temporal and spatial patterns may be significantly different.

Certain analytical choices have a particularly strong influence on the temporal and spatial distribution of air quality values observed in epidemiological studies. For example, epidemiology studies frequently use a central site monitor to represent air quality for a given area or an agglomeration of monitors across a city. Often studies note the number of monitors and source of data but provide little other information because researchers may not consider it central to the hypothesis being studied, although information regarding such monitors (e.g., location and method of measurement) is useful to understanding how risk estimates for that urban area may be influenced by particular sources such as roadways or industrial facilities. The number, location, and measurement (i.e., instrument) error of monitors are also important for interpreting uncertainty in exposure estimates because of spatial and temporal heterogeneity of pollutant concentrations—as is information regarding treatment of missing values, exceptional events, and flagged data in estimating exposure (Goldman et al. 2010; Peng and Bell 2010).

Community-level variables, including temperature and air conditioning prevalence, can modify air pollution health risks and therefore may explain differences in the relationship between pollution levels and the health responses between study populations. For example, cities with higher air conditioning prevalence tend to have smaller effect estimates for ozone and PM_2.5_ (Franklin et al. 2007; Levy et al. 2005). These and other effect modifiers can be considered formally within risk assessments or meta-analyses, provided that epidemiological studies characterize the impact of these variables and note their origin.

**Population.** Epidemiologic studies generally relate historical changes in observed air quality with a change in health risk among a particular population in specific locations. Conversely, risk assessments tend to estimate the incidence of adverse health outcomes among a population whose attributes are sometimes very different from the study population in ways that may alter the outcome of the risk assessment. Furthermore, risk assessments often model health impacts of air quality changes over the long term (U.S. EPA 2010), requiring them to project into the future the population’s size, geographic distribution, and demographic profile. For each of these reasons, detailed information regarding population age, sex, race, ethnicity, socioeconomic conditions (e.g., income, education), and spatial distribution—and how the study authors may have used these variables to adjust the risk coefficients—are each important considerations for the risk assessment. This same information is essential to risk assessments and meta-analyses because they pool results across epidemiological studies that consider populations of differing age ranges, sex, or races. Similarly, as alluded to above, although epidemiological studies sometimes adjust risk coefficients according to the key demographic (e.g., age, sex, race) or socioeconomic attributes (e.g., income, education), these results are not always presented within the paper or supplementary materials.

**Health data.** Detailed characterization of the health data used in epidemiologic studies is very important to risk assessors as they consider which end points to quantify, how to match key characteristics of the end points across populations, and how and whether to pool evidence across studies. Study authors may select *International Classification of Diseases 9th Revision* (ICD-9; WHO 1977) or *10th Revision* (ICD-10; WHO 1999) codes for a variety of reasons such as data availability and significance of findings, among others. Although this aspect of the study is not always well documented, it is very important to risk assessors as they consider which end points to quantify and how and whether to pool evidence across studies in risk assessments and meta-analyses; these points are particularly important for risk assessments that transfer effect estimates from one region to another.

In some cases, ICD codes can be inferred, but in other cases, the same descriptive end points can span a range of codes. Further, not all researchers define disease categories (e.g., “pneumonia”) with identical ICD categories, hindering comparison and synthesis across results in risk assessments or meta-analyses (Ji et al. 2011). As another example, although one study may estimate the change in respiratory hospital admissions (ICD-9 codes 460–519), another may include hospitalizations for respiratory illness (ICD-9 codes 490–492, 464–466, 480–487) (Peng et al. 2009). In some cases, it is unclear whether the selected ICD codes are an artifact of the available data or limited statistical power or a deliberate and hypothesis-driven decision. In other cases, the study may not indicate the entity that provided baseline health data, which prevents an exact replication.

Risk assessments would also benefit from a more detailed specification in the epidemiological study of key attributes of baseline health data, including whether

Death or admission was based on primary or secondary causes.
Scheduled or unscheduled hospital visits were used.
Ultimate discharges from the hospital or emergency department were fatal or nonfatal.
The emergency department visit resulted in admittance to the hospital.
The hospital or emergency department visit was one of many visits in a multiday period.
Baseline rates were age adjusted.
Baseline mortality or hospital discharge rates were spatially aggregated.
Baseline incidence rates were interpolated for locations with missing data.

Prior analyses suggest the importance of applying baseline health data that are appropriately matched to the effect coefficients used in the analysis (Hubbell et al. 2009; Wesson et al. 2010). Reporting summary statistics of health data used in epidemiological studies would also reduce uncertainties associated with matching these data in risk assessments or meta-analyses. Clearly, it will not always be possible for study authors to generate data of such detail, but generally risk assessors will benefit from more specific information.

## Encouraging Dialogue between Air Pollution Epidemiology and Risk Assessment

Laying out the key challenges in using risk estimates from epidemiological studies for risk assessments is only a tentative first step toward resolving them. An ongoing dialogue between the risk assessment and epidemiology communities is necessary both to illuminate the shared interests and needs of each community and to ensure that epidemiological findings are used appropriately. As risk assessments increase in complexity by considering multipollutant and temperature–pollutant interactions and multiple PM components and sources, the importance of this dialogue and of mechanisms to share critical information from epidemiological studies is even greater.

A few mechanisms already exist to facilitate better data sharing between epidemiologists and analysts conducting health risk assessments supporting regulatory analyses. For example, online supplements, which are accessible at either a journal or an author-specified website, can make available data, sensitivity analyses, and detailed methods that are important both to epidemiologists and to risk assessors. Placing detailed information in a supplement to the article ensures that risk assessors and epidemiologists can access key data without overwhelming readers less interested in such information; because some journals limit the size of supplementary materials, authors may wish to communicate the benefits of this information to editors. The International Society for Environmental Epidemiology published guidelines articulating the responsibilities of original investigators and those who undertake reanalyses or reinterpretation of critical epidemiological studies (Neutra et al. 2006). Likewise, a national database of risk assessments might also provide risk assessors with examples of best practices. Effective use of these tools would serve epidemiology, risk assessments, and, by extension, public health—while balancing the rights of the public and the intellectual property rights of investigators.

Although the greater availability of data and risk assessments holds great promise, such an approach would need to be weighed against the potential for these data to be misinterpreted or willfully misused. For these or any other mechanisms to be of practical use to either epidemiologists or risk assessors, an ongoing dialogue is needed between the two communities. Such a dialogue should identify the core research questions important to both communities—while fostering the most accurate risk assessments and discouraging the unintentional misuse of epidemiological findings. One way to initiate this dialogue would be to develop a series of U.S. EPA-sponsored or HEI-sponsored case studies or workshops with a goal of making clear distinctions between data or results that are likely already to exist but are not made readily available to health risk assessors, and those that may be lacking for a risk assessment because the underlying epidemiologic studies focused on different questions. The sometimes divergent aims of epidemiology and risk assessment should not preclude close collaboration between the disciplines—our scientific goals are not so far apart, and both communities stand to benefit.

## Appendix 1.

Below we summarize the information regarding the specification of effect estimates, air quality, populations, and baseline health data that we described above as being most helpful to risk assessors and epidemiologists conducting meta-analyses. This list is neither exhaustive nor intended to serve as criteria for data-reporting requirements. However, authors and editors may find this summary useful as they consider how to make information reported in epidemiological studies more accessible to other practitioners.

### Effect estimates

What type of statistical model was used?

Were sensitivity analyses performed? If so, did such analyses consider

Confounders, such as other pollutants?
Effect modifiers, such as demographic variables?

Were numerical data presented in sufficient detail, including

*t*-Statistics?
*p*-Values?
95% confidence intervals?
Standard error of central estimates?

Did the study consider combined effects? If so, was a variance–covariance matrix included?

If null or statistically insignificant results were generated, were they reported?

### Air quality

If ozone-related risk was estimated, did the study use alternate exposure metrics?

If PM-related risk was estimated, did the study summarize the particle composition?

If the study used air-quality monitor data, were the number, locations, and methods of measurement of such monitors reported?

Were community-level variables reported, including

Air conditioning prevalence?
Temperature?

### Population

Were key population characteristics reported, including age, race, sex, ethnicity, and socioeconomic status?

What is the spatial distribution of the population considered?

### Health data

For each health end point assessed, what corresponding ICD-9 or ICD-10 codes were used?

What criteria were considered when selecting ICD codes?

For the baseline health data applied:

Was the cause of admission or death based on primary or secondary causes?
Were hospital visits scheduled or unscheduled?
Were hospital discharges fatal or nonfatal?
Did the emergency department visit result in a hospital visit?
Was the emergency department or hospital visit one among many in a multiday period?
Were mortality, hospital, and emergency department visit rates age-adjusted?
Were baseline mortality rates spatially aggregated?
Were baseline rates interpolated?
